# Propagation of Fatigue Cracks in Friction of Brittle Hydrogels

**DOI:** 10.3390/gels4020053

**Published:** 2018-06-08

**Authors:** Tetsuo Yamaguchi, Ryuichiro Sato, Yoshinori Sawae

**Affiliations:** 1 Department of Mechanical Engineering, Kyushu University, 744 Motooka, Nishi-ku, Fukuoka 819-0395, Japan; ryu1ro124@gmail.com (R.S.); sawa@mech.kyushu-u.ac.jp (Y.S.); 2 International Institute for Carbon-Neutral Energy Research, Kyushu University, 744 Motooka, Nishi-ku, Fukuoka 819-0395, Japan

**Keywords:** hydrogel, friction, fatigue, wear, fracture, crack, adhesion, delamination

## Abstract

In order to understand fatigue crack propagation behavior in the friction of brittle hydrogels, we conducted reciprocating friction experiments between a hemi-cylindrical indenter and an agarose hydrogel block. We found that the fatigue life is greatly affected by the applied normal load as well as adhesion strength at the bottom of the gel–substrate interface. On the basis of in situ visualizations of the contact areas and observations of the fracture surfaces after the friction experiments, we suggest that the mechanical condition altered by the delamination of the hydrogel from the bottom substrate plays an essential role in determining the fatigue life of the hydrogel.

## 1. Introduction

Recently, hydrogels have attracted much attention of both scientists and engineers because of their unique characteristics: they have a low elastic modulus with large deformability and often exhibit extremely low surface friction [1,2,3,4,5,6]. Because they are similar to natural articular cartilages in structure and properties [7], hydrogels are expected as a candidate material for artificial articular cartilages that could overcome the drawbacks in the present hard-material-based artificial cartilages and reproduce superior characteristics of natural cartilages [8,9,10,11,12,13,14,15,16,17,18]. However, there is a serious problem that we have to resolve; the low fatigue strength of hydrogels against repetitive loadings. To tackle this problem, two different approaches can be considered: material science and mechanics approaches. The former approach is to make trials to synthesize tough hydrogels. In fact, new types of hydrogels with improved toughness have successfully been developed by material scientists in the last decade [19,20,21]. On the other hand, the latter would be to understand the basic mechanisms behind crack propagation [22,23,24,25,26,27,28] and to create novel types of hydrogels adaptive to the mechanical conditions. However, there have been few studies focusing on the fatigue behavior of hydrogels during sliding [29]; the underlying mechanisms and design principles are not well understood at present.

In this paper, we report our fundamental studies on propagation behavior of fatigue cracks of hydrogels in reciprocating friction experiments. In order to facilitate observations, we used agarose hydrogels as a typical example of brittle hydrogels. By performing in situ visualization of frictional contact and observations of fracture surfaces after the friction experiments, we investigated the mechanisms responsible for the propagation of fatigue cracks.

## 2. Results and Discussion

### 2.1. Stress-Relaxation Behavior

**Figure 1:**
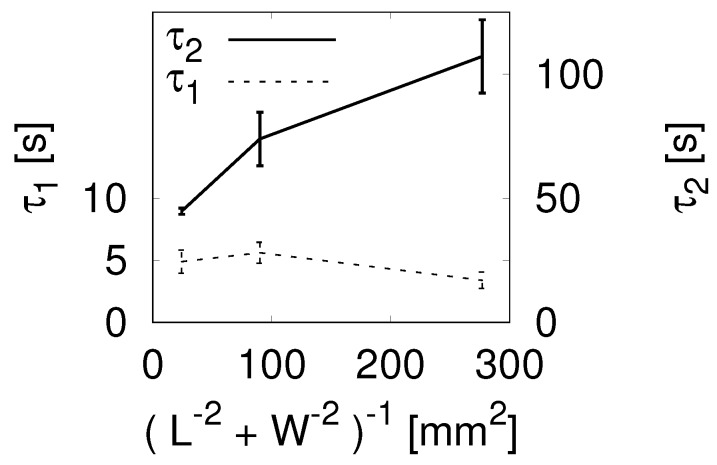
Relaxation times $\tau_{1}$ (left axis) and $\tau_{2}$ (right axis) as a function of ${\left(L^{-2}+W^{-2}\right)}^{-1}$.

First of all, we discuss the stress-relaxation behavior of agarose hydrogels on the basis of the results of the unconfined compression tests. As we mention in the experimental section, hydrogels have multiple relaxation processes because of viscoelasticity and water diffusion. In viscoelastic relaxation, the characteristic time is determined by microscopic or mesoscopic processes and is independent of the size of the hydrogel sample. On the other hand, the relaxation time due to diffusive transport should depend on its size. In order to examine the size dependence, we plotted two relaxation times $\tau_{1}$ and $\tau_{2}$ against the “system size” ${\left(1/L^{2}+1/W^{2}\right)}^{-1}$ in Figure 1 (the physical meaning of this term is discussed in Appendix A). It is clearly seen that $\tau_{2}$ was much larger than $\tau_{1}$ as well as that $\tau_{2}$ depended on the system size while $\tau_{1}$ did not. From these features, we identified $\tau_{1}$ and $\tau_{2}$ as the viscoelastic and diffusive relaxation times, respectively.

We then estimated the permeability of the polymer network from the relaxation time $\tau_{2}$. According to the stress–diffusion (diffusio-mechanical) coupling model [30] (which is equivalent with biphasic lubrication theory [31,32,33] and the modified version of Tanaka–Fillmore theory [34]), the relaxation time is described by the following equation (see further details in Appendix A):

(1) $$\tau_{2}=\frac{1}{D_{c}}\frac{1}{\pi^{2}\left(\frac{1}{L^{2}}+\frac{1}{W^{2}}\right)},$$

where $D_{c}=\kappa_{0}\left(1-\phi \right)\left(K+4/3G\right)$ is the collective diffusion constant; *K* and *G* are the osmotic and shear moduli, respectively; κ is the permeability; and ϕ is the volume fraction of polymers.

*K* and *G* were determined in the following manner. Just after we applied compression (the stress reached the maximum), the gel was regarded as an incompressible material because there was no time for water to come out of the gel. If we neglect the viscoelastic contribution to the stress (with intensity $E_{1}$), we obtain the following equation:

(2) $$G=\frac{\sigma_{z}\left(t=0\right)}{3\epsilon_{z}}=\frac{E_{\infty }+E_{2}}{3},$$

where $\sigma_{z}\left(t\right)$ and $\epsilon_{z}$ are the the compressive stress and strain respectively; $E_{\infty }$ is the relaxed modulus and $E_{2}$ is the intensity of the diffusive mode, as introduced in the experimental section. $E_{\infty }$ and $E_{2}$ are estimated from the fitting of the stress-relaxation curves. On the other hand, the normal stress at infinite time is described by

(3) $$\sigma_{z}\left(t=\infty \right)=E_{\infty }\epsilon_{z}=\frac{3GK}{K+G/3}\epsilon_{z}.$$

From Equation (3), we obtain the following relation:

(4) $$K=\frac{GE_{\infty }}{9G-3E_{\infty }}.$$

We approximate (1−ϕ)≈1 and obtain the expression for the permeability:

(5) $$\kappa =\frac{D_{c}}{K+4/3G}.$$

We calculated $D_{c}$, *K*, *G*, and κ for each sample and then averaged over all the samples. The values estimated were $D_{c}$ = 1.5 × 10^−7^ ± 9.7 × 10^−8^ m^2^/s, K = 90 ± 26 KPa, G = 66 ± 19 KPa, and κ = 8.8 × 10^−13^ ± 5.6 × 10^−13^ m^4^/Ns, respectively. Some of these values were reported by Gu et al. [35], within order-of-magnitude differences, although our values included large errors. In addition, the shear modulus *G* was in agreement with that measured in our oscillatory shear experiments.

As we discuss in the following subsections, we conducted reciprocating friction experiments between a hemi-cylindrical indenter and an agarose hydrogel block at the sliding speed V = 9.1 mm/s and the stroke S = 20 mm under the normal load $F_{N}$ ≈ 5 N. By assuming the Hertzian contact [36], we could calculate the contact length as $L_{c}=\sqrt{4F_{N}R/\pi E^{*}W}$ ≈ 0.003 m (R = 0.0145 m, $E^{*}$ ≈ 280 KPa, and W = 0.032 m being the equivalent curvature radius, the equivalent elastic modulus, and the sample width, respectively) and the maximum pressure as $P_{max}=\sqrt{E^{*}F_{N}/\left(\pi RW\right)}$ ≈ 30 KPa. From these, we discuss the time scales for the contact: the contact time was $t_{c}=L_{c}/V$ ≈ 0.3 s, and the recurrence interval of the contact was T=2S/V ≈ 4 s. In addition, the characteristic time for water to be squeezed from the contact region was given by $t_{sq}\approx L_{c}^{2}/\left(\pi^{2}D_{c}\right)$ ≈ 7 s. That is, the characteristic contact time was much shorter than the relaxation times due to water diffusion and viscoelasticity, and also much shorter than the recurrence time of the contact. This indicates that the agarose hydrogel could be regarded as an incompressible elastic material during our reciprocating friction experiments. In terms of stress, the maximum contact pressure ($P_{max}$ = 30 KPa) was well below the failure stress (≈80 KPa) of the gel, meaning that the friction experiments were performed under moderate mechanical conditions.

### 2.2. Effects of Adhesion Strength between Gel and Bottom Glass Slide

**Figure 2:**
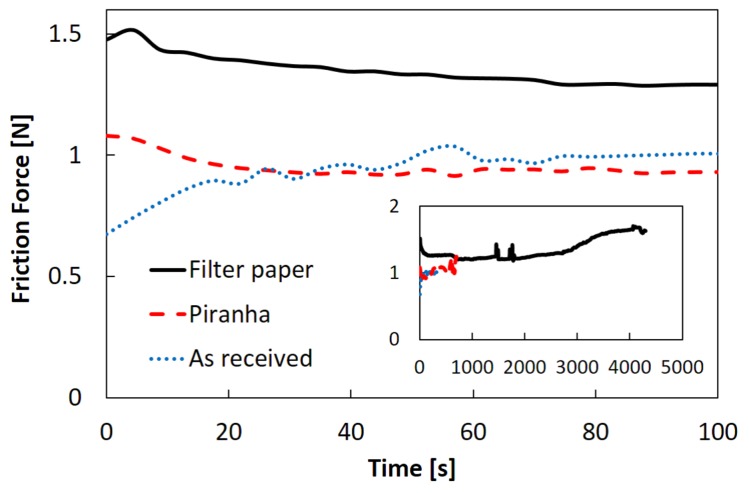
Time evolutions of friction forces for three samples of different adhesion strengths with bottom glass slide: Filter paper (solid line), Piranha (dashed line), and As received (dotted line). Main figure and inset correspond to the time evolutions of the initial 100 s and those until entire rupture of the samples, respectively. The fatigue lives were 4304 ± 56, 1457 ± 535, and 535 ± 288 s for Filter paper, Piranha, and As received samples, respectively. $F_{N}=$ 4.41 N for all samples.

We then studied the frictional behavior of the agarose hydrogels under three different adhesion-strength conditions: As received (weak; for further details refer to the experimental section), Piranha treatment (intermediate), and Filter paper (strong). In this experiment, the normal load $F_{N}$ was fixed as 4.41 N. Figure 2 shows time evolutions of the friction forces. The main figure and its inset correspond to time evolutions over the initial 100 s and those until entire rupture of the samples, respectively. As can be seen clearly, the Filter paper sample showed the largest friction forces for most of the experimental periods as well as the longest fatigue life until the entire rupture, that is, when the sample broke into two pieces. In contrast, the As received sample where the gel is adhered weakly to the glass slide broke less than 10% of the fatigue life of the gel fixed with the filter paper. For the samples with Piranha treatment, both the friction force and the fatigue life were in the middle of those values for the other two types of samples. This means that the stronger the adhesion between the gel and the glass slide, the longer the fatigue life becomes.

**Figure 3:**
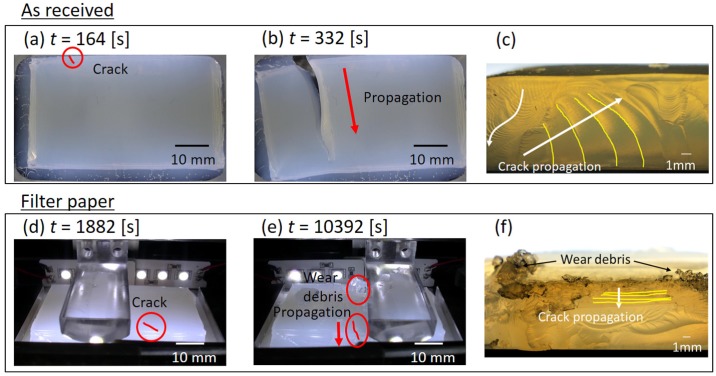
Fatigue crack behavior in As received (**a**–**c**) and Filter paper samples (**d**–**f**). (**a**,**b**) Bottom views, (**d**,**e**) side views, and (**c**,**f**) fracture surfaces after entire rupture of the samples. Small cracks are highlighted with red lines in (**a**,**d**,**e**). Arrows in (**c**,**f**) are crack paths, and yellow lines are supplementally drawn on striation patterns.

In order to understand the mechanisms, we analyzed images taken by a video camera during the friction experiments. Figure 3a,b shows the bottom views of the As received sample, and Figure 3d,e shows the side views of the Filter paper sample. It is clearly seen that, for the As received samples, a main crack was generated at the edge of the hydrogel surface and then propagated laterally in the direction perpendicular to the sliding direction. Figure 3c shows an image of the fracture surface after the friction experiment. Agarose gels behave as brittle materials, and surface cracks rather than internal cracks tend to be formed in unlubricated sliding contact. The striations, which were considered to be generated as a result of zig-zag propagation of fatigue cracks due to two different principal stress directions in reciprocating motions [37], indicated that a fatigue crack was formed at the frictional interface and propagated in the depth direction followed by the direction perpendicular to the sliding direction. On the other hand, for the Filter paper samples, a crack was formed at the edge of the sample in the same manner as for the As received sample but (as clearly seen in the fracture surface in Figure 3f) propagated slowly into the bottom, instead of following the progressive penetration and lateral propagation observed for the As received sample. It is important to note that some amount of wear debris is formed and accumulated on the frictional surface.

**Figure 4:**
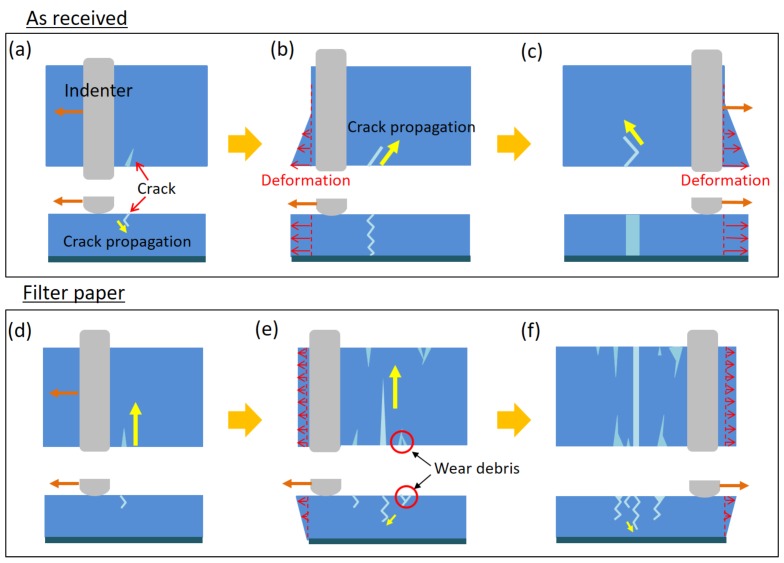
Schematic of propagation mechanisms of fatigue cracks and formation mechanisms of wear debris for (**a**–**c**) As received and (**d**–**f**) Filter paper samples.

Figure 4 schematically depicts the experimentally observed behavior. For the As received samples, after an initial crack was formed at the edge of the frictional interface, it propagated in the depth direction, as shown in Figure 4a. Once it reached the interface between the gel and the glass slide (Figure 4b), it caused sliding at the bottom gel–glass interface and also enhanced tensile deformation of the gel in the sliding direction. This large tensile deformation accelerated fatigue crack propagation in the direction perpendicular to the sliding direction (Figure 4c), leading to fast rupture. On the other hand, for the Filter paper samples, the fatigue crack propagation slowed down because large tensile deformation to continue crack propagation was suppressed as a result of the strong adhesion between the gel and the bottom glass slide (Figure 4d,e). Along with the slow crack propagation, multiple fatigue cracks were successively formed, and the coalescence of two cracks occurred, leading to wear debris formation, as shown in Figure 4e,f.

We also discuss the mechanisms responsible for generating different friction forces for the three different samples at the initial stages of the friction experiments. Because the adhesion at the gel–glass interface was strong enough for the Filter paper samples (the measured maximum shear force was 13.8 N where the indenter–gel interface was ruptured; refer to the experimental section), sliding at the bottom interface did not occur during the friction experiments, and friction forces at the indenter–gel interface were measured. On the other hand, for the As received and Piranha samples with the maximum shear forces of 5.0 and 7.8 N, respectively, the adhesion at the bottom interface was not so strong and sliding started to occur at weaker forces. These led to different friction forces measured in the friction experiments and were consistent with our observations of frictional and fracture surfaces, showing some evidence of slip at the bottom interface.

### 2.3. Effects of Normal Load

**Figure 5:**
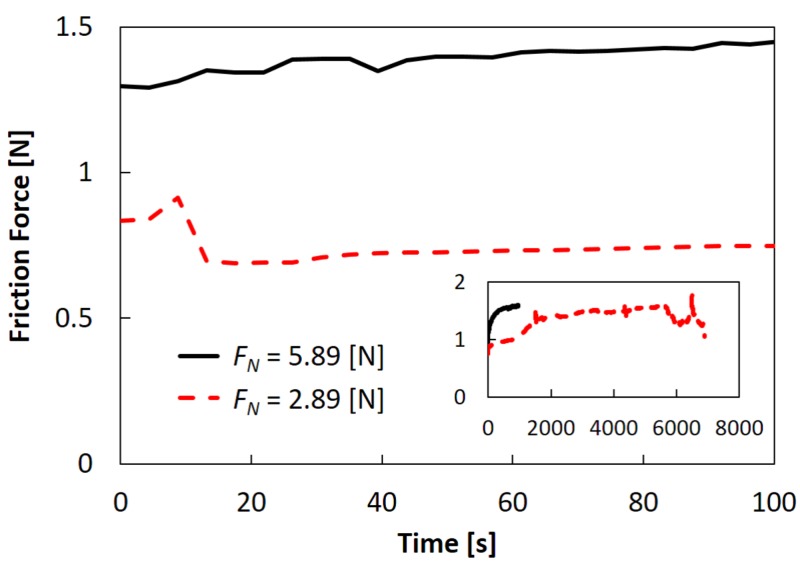
Time evolutions of friction forces under two different normal load conditions: $F_{N}$ of 5.89 N (solid line) and 2.89 N (dashed line). Main figure and inset correspond to the time evolutions of the initial 100 s and those until entire rupture of the samples. The fatigue lives were 1542 ± 938 and 4559 ± 1688 s for $F_{N}$ of 5.89 and 2.89 N, respectively. Piranha treatment was used for all samples.

**Figure 6:**
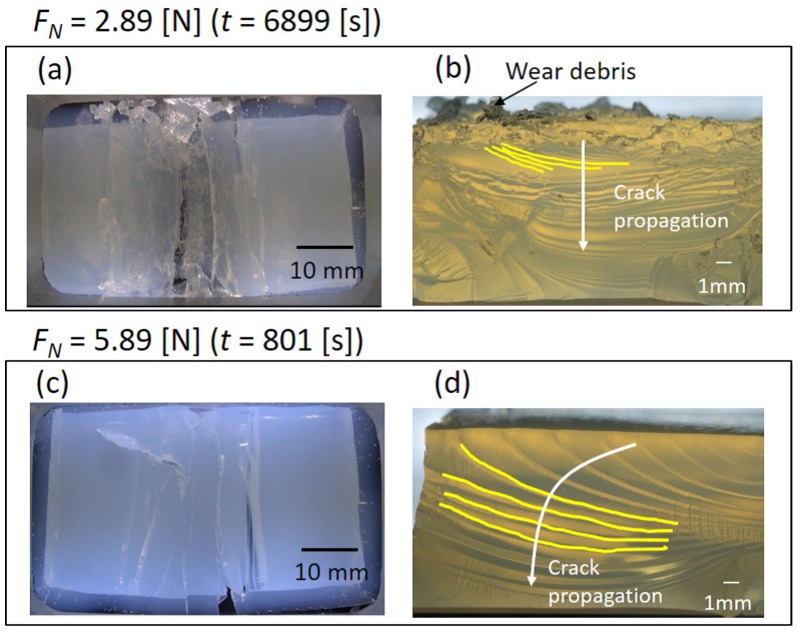
Fatigue crack behavior under two different normal load conditions: (**a**,**b**) $F_{N}$ = 2.89 N, and (**c**,**d**) $F_{N}$ = 5.89 N. Images from bottoms views (**a**,**c**) are taken just before entire rupture of each sample.

We studied the frictional behavior under two different normal load conditions. In this experiment, the adhesion condition of the bottom interface was the Piranha treatment, giving intermediate adhesion strength. The main figure and the inset of Figure 5 correspond respectively to the time evolutions of the friction forces in the initial 100 s and those until the entire rupture of the sample. As could be expected, a larger friction force was measured (roughly proportional to the normal load, i.e., giving comparable friction coefficient) and shorter fatigue life was observed for a higher normal load. Figure 6 shows bottom views just before the entire rupture of the samples (Figure 6a at t = 6899 s for $F_{N}$ = 2.89 N, and Figure 6c at 801 s for 5.89 N), and their fracture surfaces (Figure 6b,d, respectively). It is seen that a greater number of cracks were generated and slower crack propagation in the depth directions was observed for a smaller normal load. Moreover, a large amount of wear debris was formed only for the smaller normal load.

**Figure 7:**
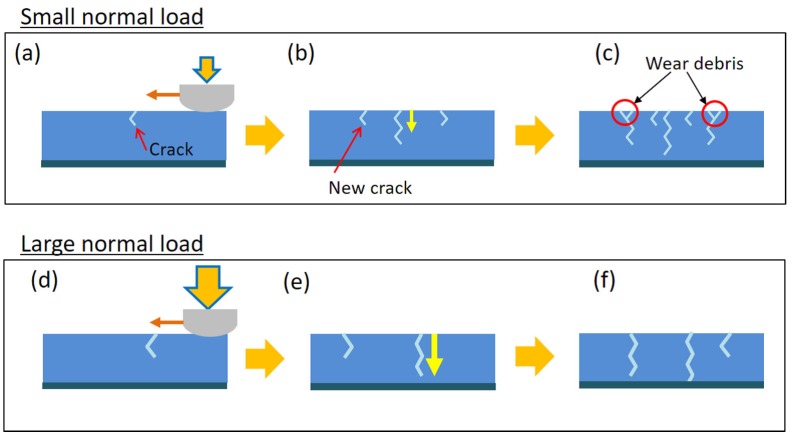
Schematic of propagation mechanisms of fatigue cracks and formation mechanisms of wear debris (**a**–**c**) for small normal loads and (**d**–**f**) for large normal loads. We note that the crack speed, i.e., time to rupture, was different between these two conditions.

The mechanisms for generating such differences are explained in Figure 7. For smaller normal load, a smaller friction force is generated. As a result, a weaker driving force for fatigue crack propagation is applied, leading to slower crack propagation and a longer fatigue life. This gives enough time for creating new cracks (Figure 7b), and if two cracks happen to merge, enclosed regions are detached from the bulk of the gel and are ejected out as wear debris. On the other hand, if the normal load is large, the fatigue cracks propagate fast enough to reach the bottom interface without creating wear debris.

### 2.4. Toward the Toughening of Hydrogels

On the basis of our results, two important points can be inferred to improve the toughness of hydrogels as frictional materials. One point is that the fixation of the gel has to be made in an appropriate manner. This leads to the slowing down of the fatigue cracks in the depth direction and a longer fatigue life, as discussed in Section 2.2. Another important point is the reinforcement of the gel along the sliding direction to support generated tensile stress and to avoid large deformation of the gel. For this purpose, the insertion of fibers into the gel matrix would be one option, as examined by Sakai and co-workers to reduce the friction of poly(vinyl alcohol) (PVA) hydrogels [38]. This is also expected to contribute to the toughening of hydrogels against friction. Both will be interesting and important topics for future studies.

## 3. Conclusions

We studied the propagation behavior of fatigue cracks during reciprocating friction experiments between a PMMA indenter and an agarose hydrogel. We found that the friction force and fatigue life were strongly influenced by the applied normal load as well as the adhesion strength at the bottom glass–gel interface. We observed the propagation speed and path of the fatigue cracks, both of which were also affected by these conditions.

## 4. Experiment

### 4.1. Sample

We prepared 3 wt % agarose hydrogels on glass slides. After dissolving agarose powder (Agarose III, Wako Pure Chemical Industries, Osaka, Japan) into hot water and stirring with an agitator, we poured the solution onto a glass slide with a rubber mold and solidified the sample in a refrigerator at 4 °C for 12 h. The sample size was *L* (length along the sliding direction) = 56 mm, *W* (width) = 32 mm, and *H* (thickness) = 10 mm. In this study, in order to examine the effects of adhesion between the gel and the bottom glass slide, we treated the glass surfaces in three different manners: one as received (hereafter called “As received”, leading to weak adhesion between the gel and glass), one with piranha treatment (“Piranha”; intermediate adhesion), and one by gluing filter paper on the glass surface (“Filter paper”; strong adhesion due to penetration of agarose solution into the filter paper before gelation). When the piranha treatment was made, a glass slide was soaked into a mixture of 30 wt % hydrogen peroxide solution (Wako Pure Chemical Industries, Japan) to sulfuric acid (Wako Pure Chemical Industries, Japan) in a ratio of 1:3 (by weight) at 80 °C for 1 h before pouring the agarose solution.

### 4.2. Friction Experiment

**Figure 8:**
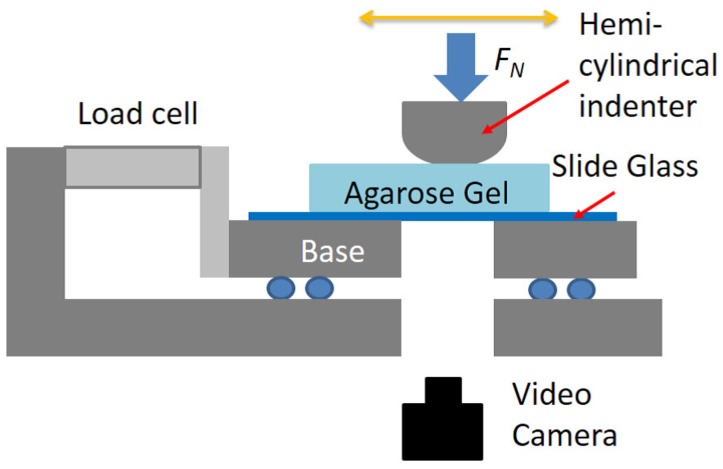
Schematic of reciprocating friction experiments.

A schematic of our experimental setup is shown in Figure 8. We conducted reciprocating friction experiments between an agarose hydrogel and a hemi-cylindrical indenter made of polymethyl methacrylate (PMMA). The curvature radius *R* and length *L* of the indenter were 14.5 and 57 mm, respectively. The indenter was placed in the direction perpendicular to the sliding direction so that it crossed both side edges of the gel sample. The sliding speed *V* was 9.1 mm/s, and the stroke *S* was 20 mm. The applied normal loads $F_{N}$ were 2.94, 4.41, and 5.89 N. We measured lateral forces acting on the bottom plate with a load cell at 100 Hz. We took the largest and smallest (with negative sign) 10 points of the friction forces per cycle, calculated average over absolute values of these 20 points, and represented it as a friction force per cycle. Under each condition, we repeated the friction experiments three times. All hydrogel samples were used as prepared, and all experiments were performed at room temperature under unlubricated conditions (without supplying additional water) with the aim to continue the experiments in a controlled manner.

### 4.3. Visualization

We observed crack propagation behavior during the friction experiments with a video camera (GZ-G5, JVC KENWOOD, Yokohama, Japan) from the bottom of the glass slide, as shown in Figure 8. Only when Filter paper samples were tested (bottom view was not available because of an opaque filter paper adhered on the glass) was observation made from the side.

### 4.4. Characterization of Mechanical Properties

Because hydrogels are soft solids composed of sparsely cross-linked polymers and a large amount of water, they exhibit mechanical relaxation due to viscoelasticity of polymer chains and diffusive transport of water molecules inside the polymer network (collective diffusion). In order to characterize such relaxation behavior, we conducted oscillatory shear experiments and unconfined stress relaxation experiments. In addition, we also conducted uni-axial compression experiments to determine the failure stress.

**Figure 9:**
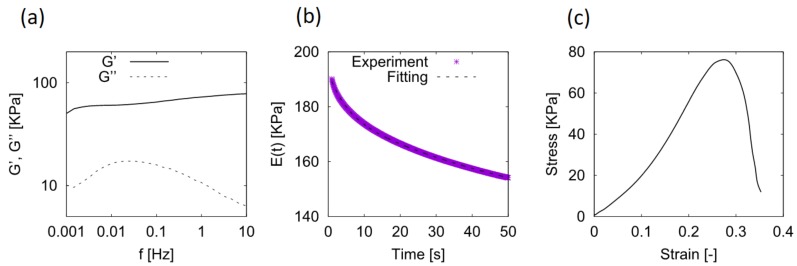
(**a**) Linear viscoelastic moduli $G^{\prime }\left(f\right)$ (storage modulus) and $G^{\prime \prime }(f)$ (loss modulus) at room temperature, (**b**) typical example of stress-relaxation behavior (l = 25 mm, w = 10 mm, h = 5 mm), and (**c**) stress–strain curve for the uni-axial compression test.

In the oscillatory shear experiments, we prepared cylindrical hydrogel samples (ϕ = 25 mm, t = 5 mm) and applied the shear of the strain amplitude γ = 0.01 and of the frequency f = 0.001–10 Hz with a parallel plate (ϕ = 25 mm) using a rheometer (MCR-301, Anton Paal, Graz, Austria). Figure 9a shows the frequency dependencies of the storage modulus $G^{\prime }\left(f\right)$ and the loss modulus $G^{\prime \prime }(f)$. The shear modulus estimated from $G^{\prime }$ was about 70 KPa. On the other hand, a clear peak in $G^{\prime \prime }(f)$ was seen around 0.03 Hz. This indicates that there existed a viscoelastic relaxation mode whose characteristic time was about 30 s.

In the stress-relaxation experiments, we prepared rectangular blocks with three different widths (L = 25 mm; W = 5, 10, 20 mm; H = 5 mm). We sandwiched each sample between two glass plates, applied unconfined uni-axial compression at V = 1 mm/s, and then fixed the displacement δ around 0.3 mm while measuring the normal force F(t) as a function of time. A typical relaxation behavior from t = 1 to 50 s is shown in Figure 9b. The relaxation function $E\left(t\right)=\sigma_{z}\left(t\right)/\epsilon_{z}=F\left(t\right)/\left(LW\right)H/\delta$ ($\sigma_{z}$ and $\epsilon_{z}$ being the normal stress and the compressive strain, respectively) seemed to have two (short and long) relaxation modes; thus we fitted the experimental data with the following equation:

(6) $$E\left(t\right)=E_{\infty }+E_{1}exp\left(-\frac{t}{\tau_{1}}\right)+E_{2}exp\left(-\frac{t}{\tau_{2}}\right),$$

where $E_{\infty }$ is the relaxed modulus, and $E_{1}$ ($E_{2}$) and $\tau_{1}$ ($\tau_{2}$) are, respectively, the intensity and the characteristic time of the shorter (longer) relaxation mode. We applied the least-squares fitting with the grid-search technique to find optimum values for $E_{\infty }$, $E_{1}$, $E_{2}$, $\tau_{1}$, and $\tau_{2}$. The obtained fitting curve is also plotted in Figure 9b and is in excellent agreement with the original curve.

In the uni-axial compression experiments, we prepared cylindrical hydrogel samples (ϕ = 10 mm, t = 5 mm). We sandwiched a sample with two glass plates and measured the normal force during compression at V = 0.1 mm/s until it broke. Figure 9c is a typical result for the stress–strain curve, showing that the failure stress and strain were about 80 KPa and 0.28, respectively.

### 4.5. Evaluation of Adhesion Strength between Gel and Bottom Slide Glass

In order to evaluate the adhesion strength between the agarose hydrogel and glass slide, we prepared gel samples in the three different manners noted above. During the gelation process, we also contacted an indenter gluing filter paper on the top surface of the agarose solution in order to fix the gel firmly against the indenter. As a consequence, when a lateral displacement of S = 20 mm was applied at V = 9.1 mm/s and $F_{N}$ = 4.41 N (in the same manner as the first cycle in the friction test), delamination occurred at the bottom glass–hydrogel interface for the As received and Piranha samples without any slip at the top indenter–hydrogel interface. Only when Filter paper samples were tested was fracture along the top interface observed. The measured maximum shear forces were 13.8, 7.8, and 5.0 N for the Filter paper, Piranha, and As received samples, respectively.

## Appendix A

Here we consider a rectangular hydrogel block with length *L*, width *W*, and height *H*. As we discussed in the previous section, viscoelastic relaxation occurs much faster than that due to the diffusive transport of water (solvent) in agarose hydrogels. Thus we assume that the gel is made of an elastic network and a large amount of water.

The constitutive relation for the polymer network is described by the following equation:

(A1) $$\sigma_{\alpha \beta }=K\frac{\partial u_{\gamma }}{\partial r_{\gamma }}\delta_{\alpha \beta }+G\left(\frac{\partial u_{\alpha }}{\partial r_{\beta }}+\frac{\partial u_{\beta }}{\partial r_{\alpha }}-\frac{2}{3}\frac{\partial u_{\gamma }}{\partial r_{\gamma }}\delta_{\alpha \beta }\right),$$

where *K* and *G* are the osmotic and shear moduli, respectively, and $u=(u_{x},u_{y},u_{z})$ is the elastic displacement.

By taking into account the diffusive transport of water, the force balances in terms of the polymer and water are described by the following equations:

(A2) $$\bar{\xi }\left(\dot{u}-v_{s}\right)=\nabla \cdot \sigma -\phi \nabla p,$$

(A3) $$\bar{\xi }\left(v_{s}-\dot{u}\right)=-\left(1-\phi \right)\nabla p,$$

where $\bar{\xi }$ is the friction constant for the relative motion between the polymer and water; $\dot{u}$ and $v_{s}$ are the local velocity of the polymer and water, respectively; ϕ is the volume fraction of the polymer; and *p* is the local isotropic pressure.

On the other hand, $\dot{u}$ and $v_{s}$ must satisfy the incompressible condition:

(A4) $$\nabla \cdot v=\nabla \cdot \{\phi \dot{u}+\left(1-\phi \right)v_{s}\}=0,$$

where v is the mean velocity. Equation (A3) can be written with the mean velocity:

(A5) $$\dot{u}-v=\kappa_{0}\left(1-\phi \right)\nabla p,$$

where $\kappa_{0}=1/\bar{\xi }$. Substituting Equation (A5) into Equation (A4), we obtain

(A6) $$\frac{\partial \omega }{\partial t}=\kappa_{0}\left(1-\phi \right)\nabla^{2}p,$$

where $\omega =\partial u_{x}/\partial x+\partial u_{y}/\partial y+\partial u_{z}/\partial z$ is the volumic strain of the polymer.

From the force balance equations (Equations (A2) and (A3)), we obtain the Poisson equation in terms of pressure:

(A7) $$\nabla^{2}p=\left(K+\frac{4}{3}G\right)\nabla^{2}\omega ,$$

and combining Equation (A7) with Equation (A6), we also obtain the diffusion equation:

(A8) $$\frac{\partial \omega }{\partial t}=D_{c}\nabla^{2}\omega ,$$

where $D_{c}=\kappa_{0}\left(1-\phi \right)\left(K+4G/3\right)$ is the collective diffusion coefficient. Thus, the problem is to solve the diffusion equation (Equation (A8)) with appropriate boundary conditions.

We consider the situation in which we apply uni-axial compression along the *z*-direction. Here we assume elastic displacements as follows:

(A9) $$\begin{array}{ccc} u_{x} & = & u_{x}\left(x,y,t\right),\\ u_{y} & = & u_{y}\left(x,y,t\right),\\ u_{z} & = & \epsilon_{z}\left(t\right)z. \end{array}$$

The stresses are described by

(A10) $$\begin{array}{ccc} \sigma_{xx} & = & \left(K-\frac{2}{3}G\right)\omega +2G\frac{\partial u_{x}}{\partial x},\\ \sigma_{yy} & = & \left(K-\frac{2}{3}G\right)\omega +2G\frac{\partial u_{y}}{\partial y},\\ \sigma_{zz} & = & \left(K-\frac{2}{3}G\right)\omega +2G\epsilon_{z}. \end{array}$$

In an unconfined compression test, the total stresses along the *x*- and *y*-directions must equal zero at any point inside the gel. From these conditions,

(A11) $$p\left(x,y,t\right)=\left(K-\frac{2}{3}G\right)\omega +2G\frac{\partial u_{x}}{\partial x}=\left(K-\frac{2}{3}G\right)\omega +2G\frac{\partial u_{y}}{\partial y},$$

and we obtain $\partial u_{x}/\partial x=\partial u_{y}/\partial y$. After some simple calculations, Equation (A10) can be written in the following manner:

(A12) $$\begin{array}{ccc} \sigma_{xx} & = & \sigma_{yy}=\left(K+\frac{G}{3}\right)\omega -G\epsilon_{z},\\ \sigma_{zz} & = & \left(K-\frac{2}{3}G\right)\omega +2G\epsilon_{z}. \end{array}$$

Because the elastic stresses must vanish on the side faces of the gel and because the compressive strain is held fixed at t=0 as $\epsilon_{z}\left(t\right)=\epsilon_{z0}$, we obtain the following equations:

(A13) $$\begin{array}{c} \omega \left(0,y,t\right)=\omega \left(L,y,t\right)=\frac{G}{K+G/3}\epsilon_{z0},\\ \omega \left(x,0,t\right)=\omega \left(x,W,t\right)=\frac{G}{K+G/3}\epsilon_{z0}. \end{array}$$

Furthermore, the pressure must vanish at t=∞, and thus

(A14) $$\omega \left(x,y,\infty \right)=\omega_{eq}=\frac{G}{K+G/3}\epsilon_{z0}.$$

Now we are ready to solve Equation (A8). We assume the solution as $\omega \left(x,y,t\right)=\omega_{eq}+g\left(x,y\right)exp\left(-t/\tau \right)$ and then substitute it into Equation (A8). We obtain

(A15) $$\nabla^{2}g\left(x,y\right)=-\frac{1}{D_{c}\tau }g\left(x,y\right).$$

By considering the boundary conditions (Equations (A13) and (A14)), we obtain the following solution:

(A16) $$\omega \left(x,y,t\right)=\frac{G}{K+G/3}\epsilon_{z0}\left\{1-\frac{\pi^{2}}{4}sin\left(\frac{\pi x}{L}\right)sin\left(\frac{\pi y}{W}\right)exp\left(-\frac{t}{\tau }\right)\right\},$$

where we take the lowest-order term only. The relaxation time τ is given by

(A17) $$\tau =\frac{1}{D_{c}}\frac{1}{\pi^{2}\left(\frac{1}{L^{2}}+\frac{1}{W^{2}}\right)}.$$

This indicates that the relaxation time is proportional to the square of the sample size (e.g., if L≫W, then $\tau \propto W^{2}$). Finally, the expression for the average compressive stress $\bar{\sigma_{zz}}$ is obtained:

(A18) $$\bar{\sigma_{zz}}\left(t\right)=\frac{G}{K+G/3}\epsilon_{z0}\left\{3K+Gexp\left(-\frac{t}{\tau }\right)\right\}.$$
